# Fluid preservation causes minimal reduction of parasite detectability in fish specimens: A new approach for reconstructing parasite communities of the past?

**DOI:** 10.1002/ece3.6379

**Published:** 2020-06-15

**Authors:** Evan A. Fiorenza, Katie L. Leslie, Mark E. Torchin, Katherine P. Maslenikov, Luke Tornabene, Chelsea L. Wood

**Affiliations:** ^1^ School of Aquatic and Fishery Sciences University of Washington Seattle WA USA; ^2^ Department of Ecology and Evolutionary Biology University of California Irvine CA USA; ^3^ Smithsonian Tropical Research Institute Panama City Republic of Panama; ^4^ Burke Museum of Natural History and Culture University of Washington Seattle WA USA

**Keywords:** historical ecology, marine fish parasites, museum collections, natural history, parasite abundance

## Abstract

Long‐term datasets are needed to evaluate temporal patterns in wildlife disease burdens, but historical data on parasite abundance are extremely rare. For more than a century, natural history collections have been accumulating fluid‐preserved specimens, which should contain the parasites infecting the host at the time of its preservation. However, before this unique data source can be exploited, we must identify the artifacts that are introduced by the preservation process. Here, we experimentally address whether the preservation process alters the degree to which metazoan parasites are detectable in fluid‐preserved fish specimens when using visual parasite detection techniques. We randomly assigned fish of three species (*Gadus chalcogrammus, Thaleichthys pacificus, and Parophrys vetulus*) to two treatments. In the first treatment, fish were preserved according to the standard procedures used in ichthyological collections. Immediately after the fluid‐preservation process was complete, we performed parasitological dissection on those specimens. The second treatment was a control, in which fish were dissected without being subjected to the fluid‐preservation process. We compared parasite abundance between the two treatments. Across 298 fish individuals and 59 host–parasite pairs, we found few differences between treatments, with 24 of 27 host–parasite pairs equally abundant between the two treatments. Of these, one pair was significantly more abundant in the preservation treatment than in the control group, and two pairs were significantly less abundant in the preservation treatment than in the control group. Our data suggest that the fluid‐preservation process does not have a substantial effect on the detectability of metazoan parasites. This study addresses only the effects of the fixation and preservation process; long‐term experiments are needed to address whether parasite detectability remains unchanged in the months, years, and decades of storage following preservation. If so, ecologists will be able to reconstruct novel, long‐term datasets on parasite diversity and abundance over the past century or more using fluid‐preserved specimens from natural history collections.

## Graphical abstract

Long‐term datasets are a prerequisite for evaluating temporal patterns in wildlife disease burdens, but historical data on parasite abundance are extremely rare. For more than a century, natural history collections have been accumulating fluid‐preserved specimens, which should contain the parasites infecting the host at the time of its preservation. Our data suggest that the fluid‐preservation process does not have a substantial effect on the detectability of metazoan parasites and that ecologists can confidently reconstruct novel, long‐term datasets on parasite diversity and abundance over the past century or more using fluid‐preserved specimens from natural history collections.

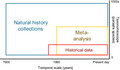

## 1 INTRODUCTION

Contemporary observations suggest that rates of wildlife disease have recently increased in frequency and magnitude (Harvell et al., 1999, 2002). Past decades have seen outbreaks of infectious disease among marine organisms, resulting in die‐offs of endangered black abalone in California's Channel Islands (Lafferty & Kuris, 1993), sea stars along the west coast of North America (Hewson et al., 2014), and pilchards in Australia (Whittington, Jones, Hine, & Hyatt, 1997). But one important question about these disease outbreaks remains unanswered: how unusual are they? Although ecologists have access to many long‐term datasets, parasites tend not to be included among these historical data (Harmon, Littlewood, & Wood, 2019). Because we lack long‐term datasets on parasite abundance, we cannot evaluate whether apparent increases in infections represent departures from historical patterns or business as usual.

One approach to reconstructing timelines of parasite abundance is to use meta‐analysis (e.g., Fiorenza et al., 2020; Ward & Lafferty, 2004; Wood, Lafferty, & Micheli, 2010), but this approach has recognized limitations. Ward and Lafferty (2004) used the rate at which disease was reported in the literature to quantify temporal trends in disease, finding increases in disease for turtles, corals, marine mammals, urchins, mollusks, and fishes between 1970 and 2001. However, a later reanalysis found that the reported positive trend for fishes was driven by an artifact and that rates of fish disease had in fact not changed over time (Wood et al., 2010), demonstrating that conclusions drawn by meta‐analysis can be susceptible to literature bias and to improper inclusion or exclusion of studies. In another meta‐analysis of the literature, Fiorenza et al. (2020) demonstrate a global increase in *Anisakis* spp. nematode abundance from 1962 to 2015. However, despite its power for resolving recent change in parasite abundance, this meta‐analysis—like others—was constrained to the time period represented in searchable online databases (i.e., the 1960s forward; Fiorenza et al., 2020, figure S2). By the 1960s, ocean ecosystems had already undergone substantial anthropogenic change, including warming, increased fishing pressure, species invasions, and reductions in pH (Lotze et al., 2006). To understand the full scope of change in parasite abundance due to anthropogenic factors, we will need data that span a greater proportion of recent human history.

**FIGURE 1:**
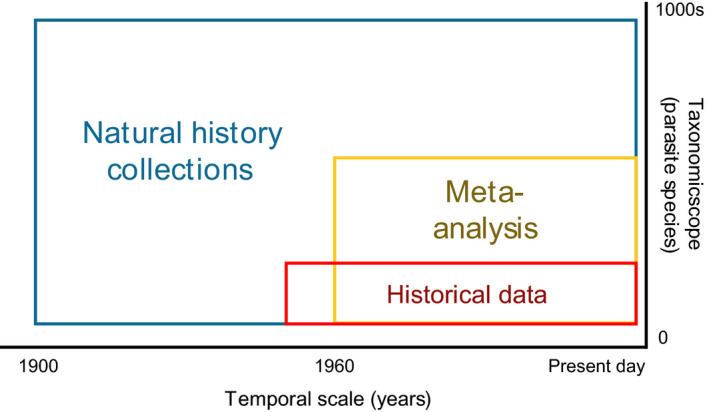
Conceptual diagram displaying the dimensions of temporal scale and taxonomic scope that can be characterized by three parasite ecology approaches. The first, historical data collected in empirical studies, involves the collection of parasite data in real time by research teams. These data are rare, are available for only a few parasite species, and tend to be limited to the latter half of the 20th century. Through meta‐analysis, researchers can summarize results across studies. Here, one is limited by the availability of accessible, published research; if using online databases, this will primarily yield studies published after 1960. Meta‐analytic techniques can only be applied to parasite species that are well‐represented in the published literature. The third method is the use of natural history collections. Collections can include specimens that are centuries old, although those accessioned prior to 1900 were probably not fixed with formalin, and we therefore do not know whether their parasite assemblages are comparable to later, formalin‐fixed samples. However, natural history collections represent many vertebrate hosts and therefore probably contain a broad swath of parasite biodiversity

Natural history collections contain specimens that could serve as useful sources of information about parasite diversity and abundance over the past century or more (Figure 1; Harmon et al., 2019). For example, Howard, Davis, Lippert, Quinn, and Wood (2019) investigated the abundance of the nematode parasite *Clavinema mariae* in English sole *Parophrys vetulus* over 84 years by counting the *C. mariae* present in fluid‐preserved fish specimens from the University of Washington Burke Museum Ichthyology Collection (UWFC). Hartigan, Phalen, and Šlapeta (2010) used natural history collections to assess whether a myxozoan parasite of an invasive cane toad was present in native Australian amphibian species before the introduction of the cane toad in 1935, and Johnson, Lunde, Zelmer, and Werner (2003) used fluid‐preserved amphibians to determine whether amphibian limb abnormalities were caused by trematode metacercaria in the past, as they are in contemporary amphibians.

Although there is substantial promise in the use of natural history collections as sources of information on parasites of the past, this approach remains to be validated (Swetnam, Allen, & Betancourt, 1999). Validation of new historical ecology approaches can be accomplished by comparison of the novel data source with established sources of information when and where they overlap, use of multiple lines of evidence, or mathematical models that test whether the results given by the novel approach are biologically and ecologically possible (McClenachan & Cooper, 2008; McClenachan, Cooper, McKenzie, & Drew, 2015; McClenachan, Ferretti, & Baum, 2012). Howard et al. (2019) provided qualitative validation for the prevalence of *C. mariae* using a historical dataset contemporaneous with specimens from natural history collections. They found that results from the two approaches (i.e., parasitological data from natural history collections versus historical datasets) both demonstrated increases in parasite abundance over time. However, Howard et al. (2019) were unable to perform more formal validation (e.g., matching natural history collection samples to historical data points and comparing the absolute abundance of parasites in the two) due to lack of natural history specimens from the appropriate times and location. Natural history collections can fill a large gap in the information available about historical rates of parasitism (Figure 1), but we must first determine whether the parasitological information stored in these collections is accurate.

Here, we address this research gap with an experimental study testing how the fluid‐preservation process used by natural history collections affects the detectability of metazoan parasites in preserved specimens. Many natural history collections use 10% formalin fixation followed by long‐term storage in 70% ethanol, which could affect parasite detectability and lead to dislodging of ectoparasites (Kvach, Ondračková, Janáč, & Jurajda, 2018). The fluid‐preservation process dehydrates the specimen and alters the coloration and physical properties of the host's tissues (Gaston, Jacquemin, & Lauer, 2013; Waller & Eschmeyer, 1965), changes that could make finding parasites in the tissues more difficult; for example, color differences between parasite and host tissue could be eliminated, and the fixation process might bind parasites more firmly to the host tissues in which they are embedded.

Kvach et al. (2018) examined the use of different field preservation techniques (fresh, frozen, 70% ethanol preservation, 4% formaldehyde fixation) on the detectability of parasites in two freshwater fish hosts (*Perca fluviatilis* and *Rhodeus amerus*). Our study differs from Kvach et al. (2018) in that we address the impact on parasite detectability of the preservation protocol used by natural history collections, which involves fixation in 10% buffered formalin followed by storage in 70% ethanol (Jenkins et al., 2014). This procedure is used by many museum collections staff because they deem it the optimal procedure for preservation of fish tissue; it might also provide good preservation of parasite tissue. We designed our experiment to test whether this procedure results in biases in the detectability of parasites in fluid‐preserved natural history specimens.

To understand how the preservation process affects the detectability of metazoan parasites, we recreated the fixation and preservation process for a variety of fish species and compared parasite abundances in experimentally preserved fish (i.e., fish that are frozen at collection, thawed at a later date, and fluid‐preserved) to control fish (i.e., fish that are frozen at collection and thawed at a later date). We hypothesized that there would be a loss of parasite detectability due to the preservation process. Specifically, we expected that mobile ectoparasites would be dislodged and lost through the preservation process as fish are moved and handled, but that permanently anchored ectoparasites would persist through the preservation process. We expected that endoparasites would generally be preserved with the host tissues, with the degree of degradation in detectability depending on the taxonomic group to which the parasite belongs. For example, with their hard cuticles, nematodes would be likely to persist through the preservation process. Adult trematodes, adult cestodes, and adult acanthocephalans might be harder to identify after fixation, due to degradation of structures used for taxonomic diagnosis and loss of translucency. Trematode metacercariae and encysted cestodes might be harder to detect after fixation, as metacercaria and cysts are often embedded in host tissues and fixation could make host tissues less pliable and more opaque, interfering with visual parasite detection.

We tested these hypotheses by performing parasitological dissection of fish specimens in two treatments: one in which we experimentally recreated the fluid‐preservation process used by natural history collections (i.e., fish were frozen at collection, thawed at a later date, fixed, and fluid‐preserved), and a control (i.e., fish were frozen at collection and thawed at a later date). Preservation causes a considerable amount of chemical change in a specimen, with additional change occurring over the long periods during which specimens are stored in natural history collections. Our study aimed to address only the effects of initial fixation and transfer to preservative; long‐term experiments are needed to address whether parasite detectability remains unchanged in the months, years, and decades following preservation. In the absence of parasite detectability changes during these two stages (i.e., preservation and long‐term storage), we would conclude that natural history collections could be an accurate and useful source of information about parasite abundance in the past.

## 2 METHODS

### 2.1 What constitutes a long‐term dataset in the disease ecology literature?

First, we were interested in understanding the extent to which long‐term datasets are available in the literature, with the aim of identifying temporal gaps that could be filled by parasitological dissection of fish specimens held in natural history collections. To determine the average length of a long‐term dataset on the abundance of fish parasites, we performed a meta‐analysis. We searched ISI Web of Science for articles on long‐term studies of fish parasites in the natural environment. We used the search string *TS = ((long‐term OR long term) AND parasite AND fish)* on 30 March 2020, which returned 285 potential articles. We then screened the titles and abstracts of these articles for relevance to our question. To be included, each study had to meet the following criteria: (a) contain annual or nearly annual (i.e., >50% of temporal period had observations) observations of parasite abundance or prevalence in fishes (i.e., a study comparing two observations of parasite abundance 40 years apart is not eligible), (b) describe observations, not the results of an experimental manipulation, (c) contain data on parasitic infections of marine or freshwater fishes, not invertebrates, (d) contain data that do not arise from parasitological dissection of natural history collections, (e) include the phrase “long term” or “long‐term” in the title or abstract as a descriptor of the study (e.g., “long‐term speciation” would not meet the criteria), and (f) contain data from wild fish, not aquaculture‐reared fish. This winnowing yielded 25 articles. From each of these articles, we extracted the length of the study in years to calculate summary statistics.

### 2.2 Study species

We used three species of marine fish for our experiment. Two species, Walleye Pollock *Gadus chalcogrammus* and Eulachon *Thaleichthys pacificus*, were provided to us by UWFC. Both fish species were collected by National Oceanic and Atmospheric Administration research cruises in Alaska, frozen, and shipped to UWFC, where they were stored frozen as they awaited cataloging. The Walleye Pollock were collected from the Bering Sea and Gulf of Alaska between 2000 and 2002, and the Eulachon were collected from Shelikof Strait in the Gulf of Alaska in March of 2002. On 11 and 12 May 2018, we collected English sole *Parophrys vetulus* by otter trawl in Port Madison, WA, in conjunction with the Fisheries Ecology course offered by the School of Aquatic and Fisheries Sciences at the University of Washington. English sole were euthanized, placed on ice, and frozen within 6 hr of collection. These three species of fish represent a variety of trophic strategies (suspension feeder, benthic predator, and pelagic predator, respectively), life history (i.e., short lived [Eulachon] and long lived [English Sole and Walleye Pollock]), habitats (benthic [English Sole] and pelagic [Walleye Pollock and Eulachon]), and body plans (i.e., flat fish [English Sole] and fusiform [Walleye Pollock and Eulachon]). Fish traits might influence the parasites that the fish can be infected with or the effects of preservative on fish tissues and thus having a diversity of fish traits allows more robust conclusions regarding the effects of preservation on parasite detectability. In total, we collected 99 English Sole, 109 Walleye Pollock, and 70 Eulachon.

### 2.3 Power analysis

We sought to ensure that we had sufficient statistical power to detect moderate differences between treatments, so that if no effect of treatment was found, we could confidently conclude that there were no large differences between the treatments (i.e., so that we could rule out the possibility that the experiment was insufficiently powered to detect differences between treatments). To determine the minimum sufficient sample size, we conducted a power analysis using simulated data. In the simulation, we generated data based on a negative binomial distribution—a distribution that accurately represents most parasite populations (Shaw, Grenfell, & Dobson, 1998)—for the two treatments. We varied the sample size per treatment and the difference between means (and therefore variance, since variance is based on the mean in a negative binomial distribution) to determine the level of power needed to detect differences between treatments. We assumed that a small effect size would be equal to 0.2, a moderate effect size would be equal to 0.5, and a large effect size would be equal to 0.8 (Cohen, 1988). Using the simulated data, we then created and ran generalized linear models using a negative binomial error distribution. The first model included the effect of treatment, while the second model was a null model. After running the two models, we compared them using Akaike's information criterion (AIC) and recorded whether the treatment model was the better model (i.e., greater than 2 AIC units lower than the null model) for each simulation. We repeated the simulation 1,000 times for each sample size and difference between means and used the proportion of times the treatment model was supported by AIC to calculate power for each combination of sample size, mean, and difference between means.

### 2.4 Experimental test of the effect of preservation on parasite detectability

We took an experimental approach to assess whether and how the preservation process affects the detectability of parasites. For each of the three fish species, individuals were randomly assigned to one of two treatments using a stratified design. Prior to randomly assigning fish to a treatment, fish were visually paired according to length within each species. Then, an individual from each pair was randomly assigned to the preservation treatment by a coin toss. This stratified random design equalized host size, a potential driver of parasite abundance, between treatments, and ensured that the mean, median, and range of host body sizes were similar between the two treatments.

Two treatments were included in this experiment. In the control group, frozen fish were thawed and dissected, and their parasites identified. In the treatment group, frozen fish were thawed and preserved according to methods used at the University of Washington Fish Collection (UWFC). We followed the preservation protocol used by the UWFC, by first placing completely thawed fish in 10% buffered formalin solution until the fish was fixed (absolute amount of time was variable due to variation in fish body size, range = 9–16 days). A fish was considered to be fixed when its tissues had become firm (particularly around the abdominal cavity) but retained some pliability. Overfixation and decalcification are possible if fish are left in formalin beyond the time required to fix all the tissues. To prevent this from happening, fish were monitored during the preservation process at least every three days. Once the fish were fully fixed, they underwent two consecutive freshwater rinses, each lasting 24 hr. After this, the fish were placed directly in 70% ethanol, where they remained for at least three days prior to dissection. Across the entire preservation process, care was taken to ignore parasites that could be dislodged during the preservation process, to mimic actual preservation conditions in collections. After preservation was complete, fish were dissected, and their parasites identified.

### 2.5 Parasitological dissection

To avoid confounding the effects of preservation and dissection method, we used a consistent dissection protocol across both treatments. This could result in lower overall detectability (across both treatments) for parasites found in muscle, since candling was used rather than a muscle squash to avoid excessive destruction of the musculature of specimens (Levsen, Lunestad, & Berland, 2005)—a variation in dissection protocol that is often used for museum specimens to preserve external morphology. Methods for all other tissues follow standard parasitological techniques (see below). We compared parasite abundance between the two treatments to assess the effects of preservation on detectability for each parasite taxon detected.

For each specimen, we made a ventral incision from the anus to the gill isthmus. We then removed all viscera for examination. The viscera were separated by organ and individually squashed between two glass plates and examined for parasites. Gills from the right or blind side of the fish were removed, placed in a vial with artificial seawater (for control group) or 70% ethanol (for preservation treatment), shaken to free parasites from the gills, and examined under a dissecting microscope. External surfaces and the buccal cavity of the fish were also examined for ectoparasites. The specimen was then spread open along its ventral incision and placed over a strong light to examine the flesh and skin for parasites. All examinations were conducted under a stereomicroscope to best capture the entire parasite burden and not simply the large‐bodied parasite taxa, and all parasites were identified to the lowest possible taxonomic level using standard parasite identification keys.

### 2.6 Comparing host body size between treatments

To ensure that there were no systematic differences between treatments in fish length, we used linear mixed‐effects models (LMMs) to compare standard lengths, using experimental treatment as a fixed effect and collection locality as a random effect, for each of the three species.

### 2.7 Comparing abundance of each parasite taxon between treatments

To test for differences in mean abundance of each parasite taxon between treatments, we used generalized linear mixed‐effects models (GLMMs) for each host–parasite pair with an overall prevalence greater than five percent. In each model, we included standard length of the host and treatment as fixed effects. Collection tow ID was included as a random effect for English sole only. Models used a negative binomial error structure with a log‐link and were implemented in glmmADMB (Fournier et al., 2012; Skaug, Fournier, Bolker, Magnusson, & Nielsen, 2013). The resulting p‐values were Bonferroni‐corrected to control for false positives.

### 2.8 Comparing abundance of each parasite group between treatments

We were not only interested in whether there were significant differences between treatments in the abundance of individual parasite taxa; we also wanted to test whether there were tendencies toward over‐ or under‐representation in the preservation treatment across all parasite taxa, across all parasite taxa within life stage (i.e., adult versus larva), and across all parasite taxa within higher‐order taxonomic groupings of parasites. That is, we sought to pool replication across parasite taxa to rigorously test whether there were consistent detectability differences between the preservation treatment and control group across groups of parasites that share characteristics in common. We used a meta‐regression approach to assess the responses of parasites to treatment across parasite taxa. For effect‐size estimates, we used regression coefficients for the effect of treatment on abundance of each parasite in each host, extracted from the GLMMs described above. All analyses were implemented in *metafor* (Viechtbauer, 2010; after Wood, Sandin, Zgliczynski, Guerra, & Micheli, 2014). We first calculated a cumulative effect size across all host–parasite combinations, using a fixed‐effects model weighted by the inverse of the variance for each effect size. We tested our hypotheses about how different parasite taxa and life stages would respond to preservation with a single meta‐analytic fixed‐effects general linear model, by including the moderators: parasite taxonomic grouping (Acanthocephala, Cestoda, Nematoda, Hirundea, and Trematoda) and parasite life stage (larval and adult).

### 2.9 Comparing detectability of parasites across fish organs

Not only were we interested in how fixation and preservation influenced parasite detectability across parasite taxa and life stages; we were also interested in how detectability differences between the treatments varied across host tissues. To test this, we used a meta‐regression approach, in which we extracted the effect size and error estimates from our individual parasite taxon GLMMs outlined above. In our meta‐regression model, we correlated the effect sizes to the organs where parasites were found (i.e., body cavity, buccal cavity, heart, gills, stomach, intestine, pyloric ceca, fins, muscle, liver, kidney, gonads, and eyes), on a presence/absence basis. This allowed us to determine whether the influence of fixation on parasite detectability was modulated by the host tissue in which a parasite was found.

## 3 RESULTS

### 3.1 What constitutes a long‐term dataset in the disease ecology literature?

We found that published fish parasite studies that are described by the authors as “long‐term” have an average length ± standard error of 12.4 ± 1.9 years, with a minimum of 1 year, a median of 11 years, and a maximum of 41 years (Table S1).

### 3.2 Power analysis

**FIGURE 2:**
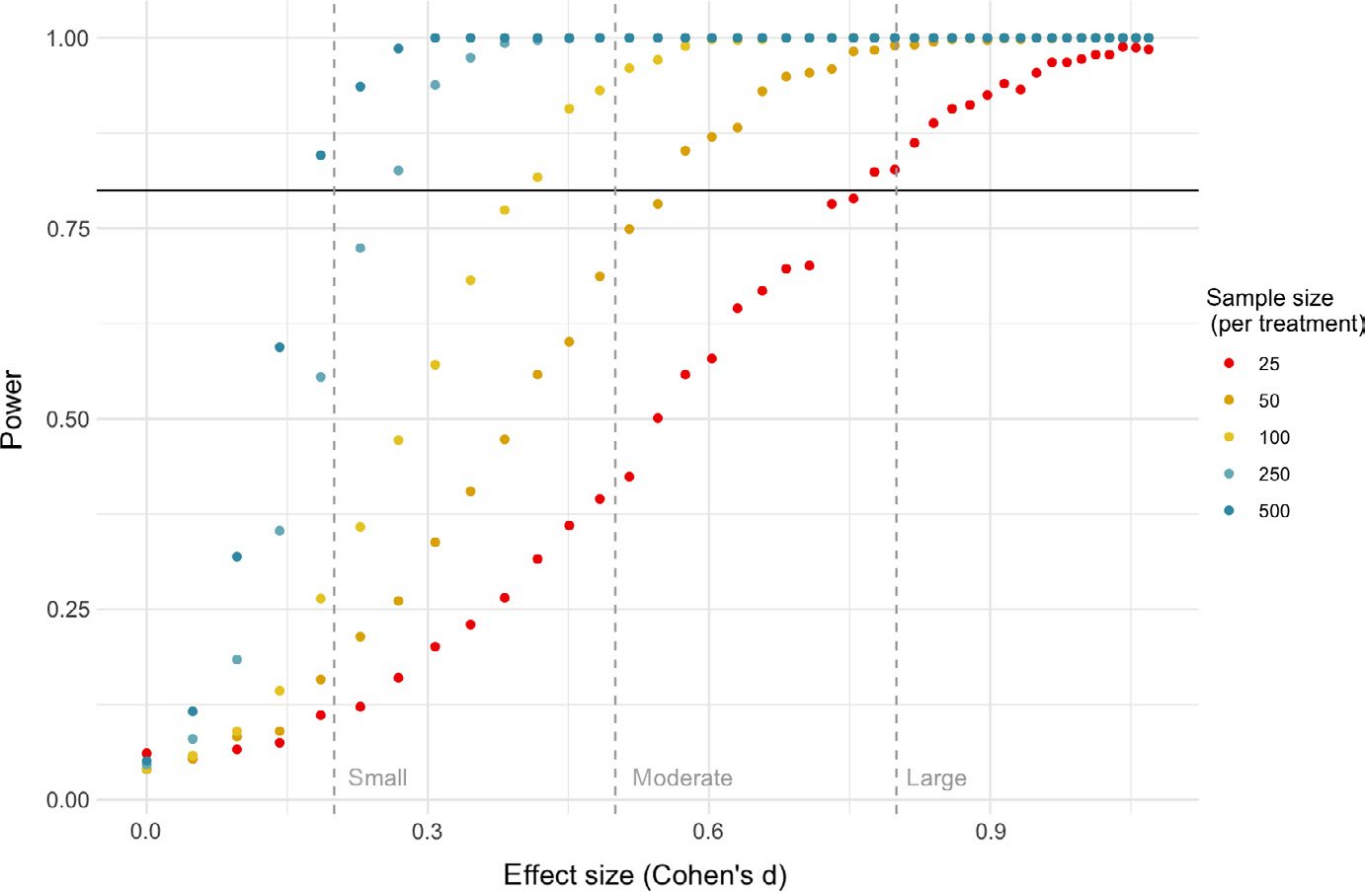
In calculations that randomly simulated the distribution of parasites across fish individuals, power to detect a difference in parasite abundance between control group and preservation treatments increased with increasing sample size and increasing effect size. Power to detect a moderate effect (effect size = 0.57) reached 0.80 at a minimum sample size of 50 individuals per treatment per fish species. Vertical dashed lines correspond to the different “levels” of effect sizes based on Cohen (1988). The horizontal black line represents a power of 0.80. In order to achieve adequate power to detect small effect sizes, approximately 500 fish would be needed per treatment–species combination

We found that a sample size of 50 fish per treatment per fish species (100 fish total per fish species) would give us the power to detect a moderate effect size of 0.57 at least 80% of the time, which is equivalent to a difference between treatments of 1.5 parasites per host. A sample size of 25 fish per treatment per fish species (50 fish total per fish species) would give us the power to detect a large effect size of 0.78 at least 80% of the time, which is equivalent to a difference between treatments of 2.3 parasites per host (Figure 2).

### 3.3 Experimental test of the effect of preservation on parasite detectability

We dissected 298 fish individuals of three species and found a total of 15,239 parasites. These parasites occurred in 59 host–parasite pairs (21 parasite taxa in English Sole, 24 in Walleye Pollock, and 14 in Eulachon). Of these 59 host–parasite pairs, 27 pairs had prevalence greater than 5% and were therefore included in analyses. These 27 pairs included adult and larval trematodes, adult and larval cestodes, adult acanthocephalans, adult and larval nematodes, and adult leeches.

### 3.4 Comparing host body size between treatments

There were no significant differences between treatments in host standard length (English sole[preserved]: estimate ± *SE* = −5.097 ± 6.788, *t*_92_ = –0.751, *p* = .455; eulachon[preserved]: estimate ± *SE* = –4.4176 ± 4.8474, *t*_56_ = –0.911, *p* = .3660; Walleye Pollock[preserved]: estimate ± *SE* = –3.287 ± 2.517, *t*_104_ = –1.306, *p* = .19446; Table 1).

**TABLE 1:** Number of individuals examined, range of standard lengths, mean of standard lengths, and *SE* of standard lengths, for hosts of each species (Walleye Pollock *Gadus chalcogrammus* from Alaska, USA*,* Eulachon *Thaleichthys pacificus* from Alaska, USA*,* and English Sole *Parophrys vetulus* from Washington, USA) in each treatment (preservation treatment versus control group)

Host species	Treatment	*n*	Range of SL	Mean SL	*SE* SL
Walleye Pollock *Gadus chalcogrammus*	Preservation	54	95–225	144	4.73
	Control	55	87–204	148	4.81
Eulachon *Thaleichthys pacificus*	Preservation	27	113–199	166	3.62
	Control	43	128–205	168	2.80
English Sole *Parophrys vetulus*	Preservation	51	83–240	154	5.54
	Control	48	81–239	156	5.63

### 3.5 Comparing abundance of each parasite taxon between treatments

**FIGURE 3:**
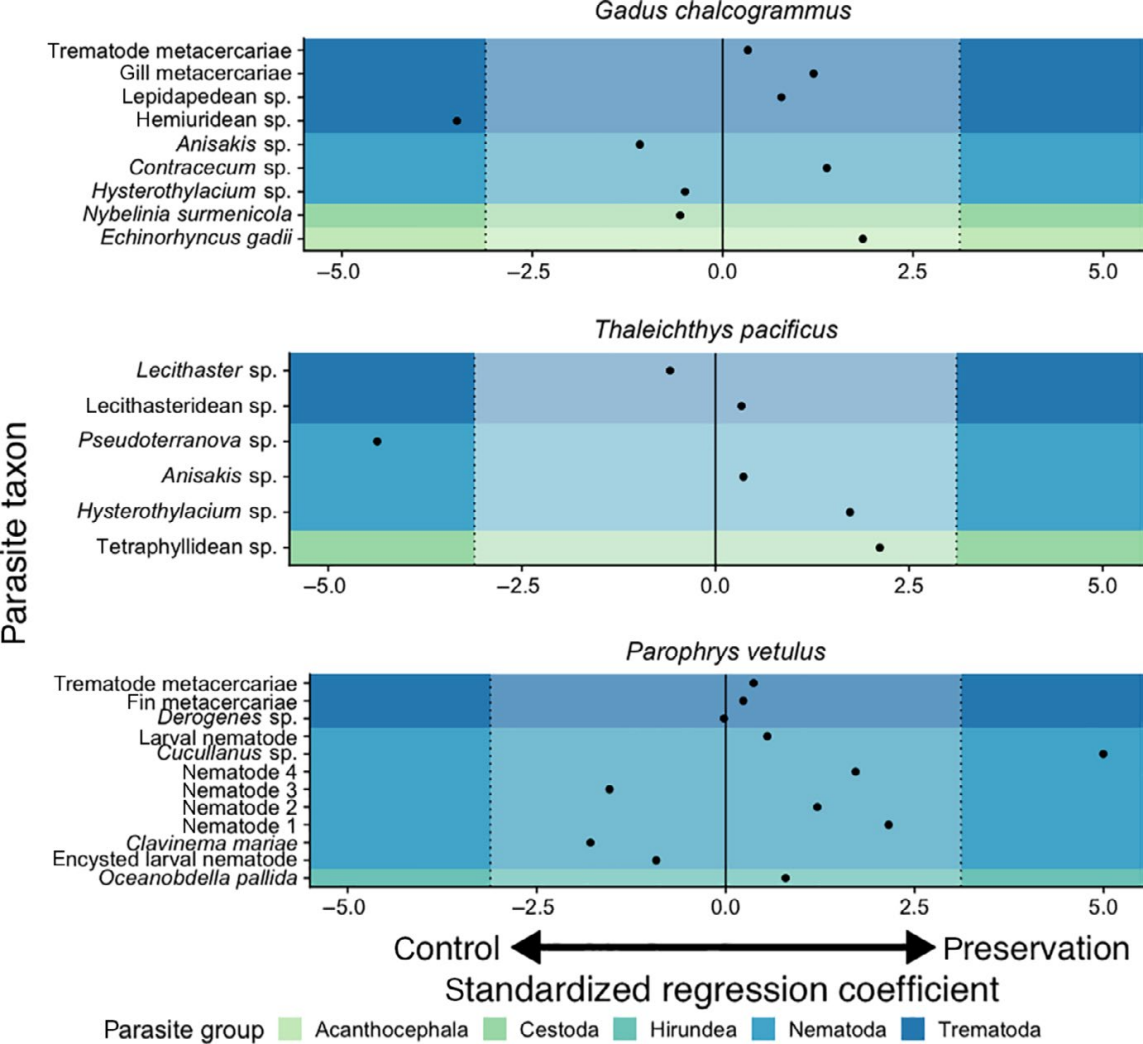
Standardized regression coefficient (*z*‐score) for the effect of preservation on the detectability of individual parasite taxa across the three host species. Negative values indicate parasite taxa where more individuals were detected in the control group than in the preservation treatment. Positive values indicate parasite taxa where more individuals were detected in the preservation treatment than in the control group. Values > 3.1 indicate enhanced detection in the preservation treatment after Bonferroni corrections. Values < –3.1 indicate decreased detection of the parasite taxa in the preservation treatment after Bonferroni corrections

Across the 27 host–parasite pairs (Table 2), we found that 24 of the 27 pairs showed no statistically significant difference in abundance between treatments. We also detected three host–parasite pairs that were significantly different in mean abundance between treatments after Bonferroni correction. Of these, one parasite taxon was more abundant in the preservation treatment compared to the control group (*Cucullanus* sp.) and two were less abundant in the preservation treatment compared to the control group (*Pseudoterranova* sp. and hemiuridean sp. trematode; Figure 3).

**TABLE 2:** Prevalence (% of hosts infected), mean abundance (mean number of parasites per host), and standard error of mean abundance for all parasites observed in >5% of individuals of each fish species (Walleye Pollock *Gadus chalcogrammus* from Alaska, USA*,* Eulachon *Thaleichthys pacificus* from Alaska, USA*,* and English Sole *Parophrys vetulus* from Washington, USA) across two treatments (preservation treatment versus control group)

Host species	Parasite taxon	Prevalence	Mean abundance	*SE* mean abundance
Walleye Pollock *Gadus chalcogrammus* (*n* = 109)	Trematode metacercariae	0.1101	0.5505	0.2890
	Gill metacercariae	0.6147	14.2385	6.0995
	Lepidapedean sp.	0.2294	1.4128	0.3280
	Hemiuridean sp.	0.1927	0.7890	0.1768
	*Anisakis* sp.	0.3211	0.9174	0.1576
	*Contracecum* sp.	0.3211	1.2661	0.2047
	*Hysterothylacium* sp.	0.3394	2.1835	0.4434
	*Nybelinia surmenicola*	0.0734	0.1468	0.0502
	*Echinorhynchus gadii*	0.1560	0.4587	0.1370
Eulachon *Thaleichthys pacificus* (*n* = 70)	*Lecithaster* sp.	0.0857	0.4857	0.2620
	Lecithasteridean sp.	0.1143	0.3714	0.1368
	*Psuedoterranova* sp.	0.6429	4.8571	0.8105
	*Anisakis* sp.	0.0857	0.2000	0.0829
	*Hysterothylacium* sp.	0.2571	0.9143	0.2327
	Tetraphyllidean sp.	0.1000	0.2000	0.0722
English Sole *Parophrys vetulus* (*n* = 99)	Trematode metacercariae	0.0606	0.5252	0.2480
	Fin metacercariae	0.3737	204.5455	49.0760
	*Derogenes* sp.	0.0505	0.1212	0.0561
	Larval nematode	0.2525	4.4040	1.0632
	*Cucullanus* sp.	0.8687	24.6667	3.2619
	Nematode 4	0.1818	2.0606	0.7095
	Nematode 3	0.0909	0.2222	0.0754
	Nematode 2	0.0909	0.2828	0.1075
	Nematode 1	0.0808	0.2424	0.0874
	*Clavinema mariae*	0.7778	28.6262	4.1490
	Encysted larval nematode	0.1111	0.2828	0.0862
	*Oceanobdella pallida*	0.3333	0.9899	0.1684

### 3.6 Comparing abundance of each parasite group between treatments

**FIGURE 4:**
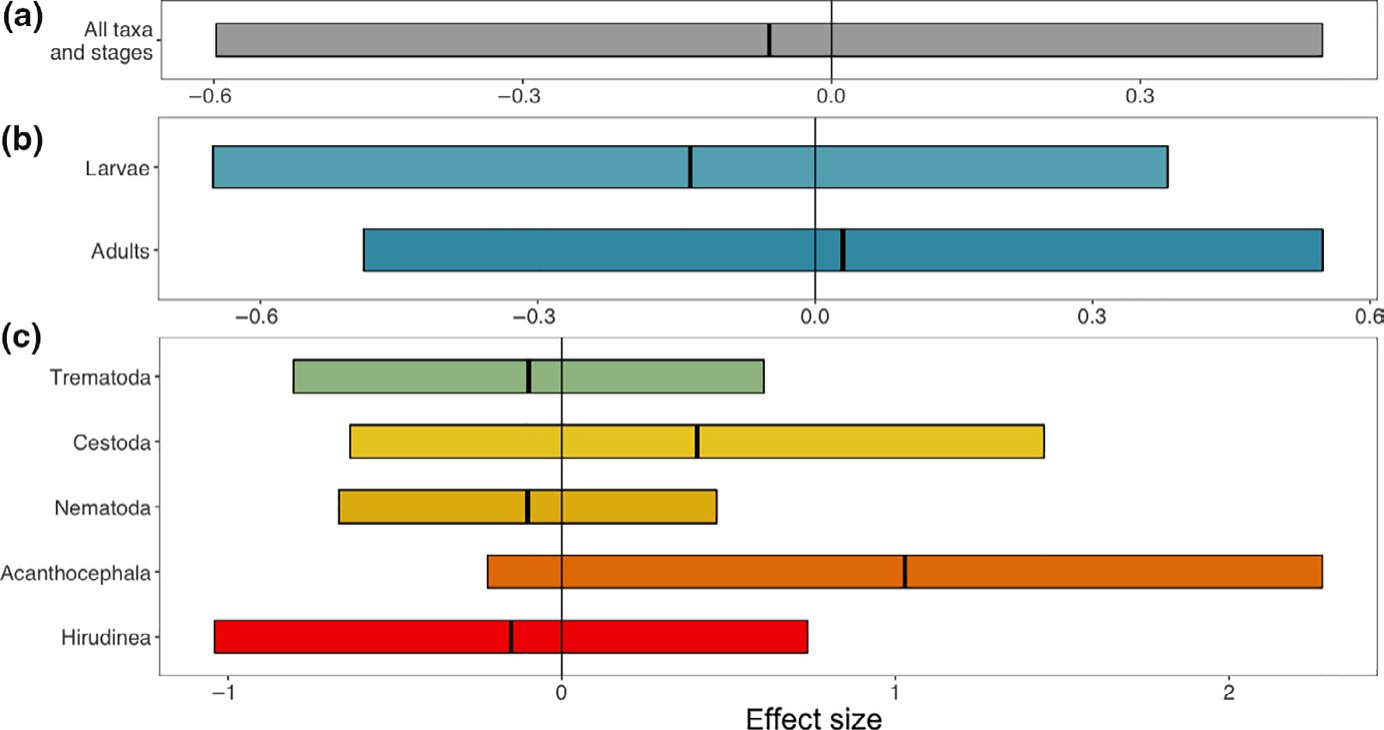
Meta‐regression estimates for the effects of preservation on mean abundance of parasites. Estimates of the effect sizes come from meta‐regression models testing the hypotheses of whether there is (a) an overall effect of preservation on detectability, (b) that detectability is moderated by parasite life stage, and (c) that detectability is moderated by parasite taxonomic group. Positive values indicate higher abundance in preserved fish while negative values indicate higher abundance in control fish. Estimates are shown with 95% confidence intervals

Using the GLMM model estimates (from *Comparing abundance of each parasite taxon between treatments*, above), we ran a set of meta‐regression models to determine whether there were consistent effects of preservation on detectability across all parasites, among parasite life stages (larval or adult), and among taxonomic groups (Trematoda, Cestoda, Nematoda, Acanthocephala, Hirudinea). We found that there were no consistent differences in detectability between treatments across all parasitic taxa, nor across life stage, nor across taxonomic group (Table 3a and Figure 4).

**TABLE 3:** Results of general linear models for meta‐analysis

Parameter	Estimate	*SE*	*z*	*p*
(a) Model 1: Effect of parasite life stage and higher‐order taxonomic groups				
Intercept	1.0202	0.6317	1.6150	.1063
Stage [larvae]	−0.0929	0.2151	−0.4319	.6658
Group [Cestoda]	−0.5501	0.7440	−0.7394	.4597
Group [Hirudinea]	−1.1316	0.6990	−1.6188	.1055
Group [Nematoda]	−1.0686	0.6037	−1.7701	.0767
Group [Trematoda]	−1.0768	0.6212	−1.7332	.0831
(b) Model 2: Effect of parasite location within host				
Intercept [muscle]	0.7299	0.9148	0.7979	.4249
Location [body cavity]	0.0867	0.5871	0.1477	.8826
Location [pyloric cecae]	−0.0374	0.4709	−0.0795	.9366
Location [stomach]	0.3773	0.4573	0.8251	.4093
Location [intestine]	−0.6503	0.8295	−0.7840	.4331
Location [kidney]	0.3134	0.7092	0.4419	.6585
Location [heart]	−2.3568	0.8541	−2.7594	**.**0058
Location [fins]	−0.8168	1.0507	−0.7774	.4369
Location [liver]	0.1664	0.5324	0.3125	.7546
Location [gonad]	−0.4482	0.6384	−0.7021	.4826
Location [gills]	0.0876	0.8885	0.0986	.9215
Location [eye]	−0.9057	1.0865	−0.8336	.4045
Location [buccal cavity]	−0.7666	0.6353	−1.2067	.2276

Note

(a) Model 1: Test for residual heterogeneity, *Q*_T_ = 80.8714, *df* = 21, *p*‐value < .0001; Test of moderators, *Q*_M_ = 4.9086, *df* = 5, *p*‐value = .4271.

(b) Model 2: Test for residual heterogeneity, *Q*_T_ = 41.1254, *df* = 14, *p*‐value = .0002; Test of moderators, *Q*_M_ = 40.1132, *df* = 12, *p*‐value < .0001.

### 3.7 Comparing detectability of parasites across fish organs

**FIGURE 5:**
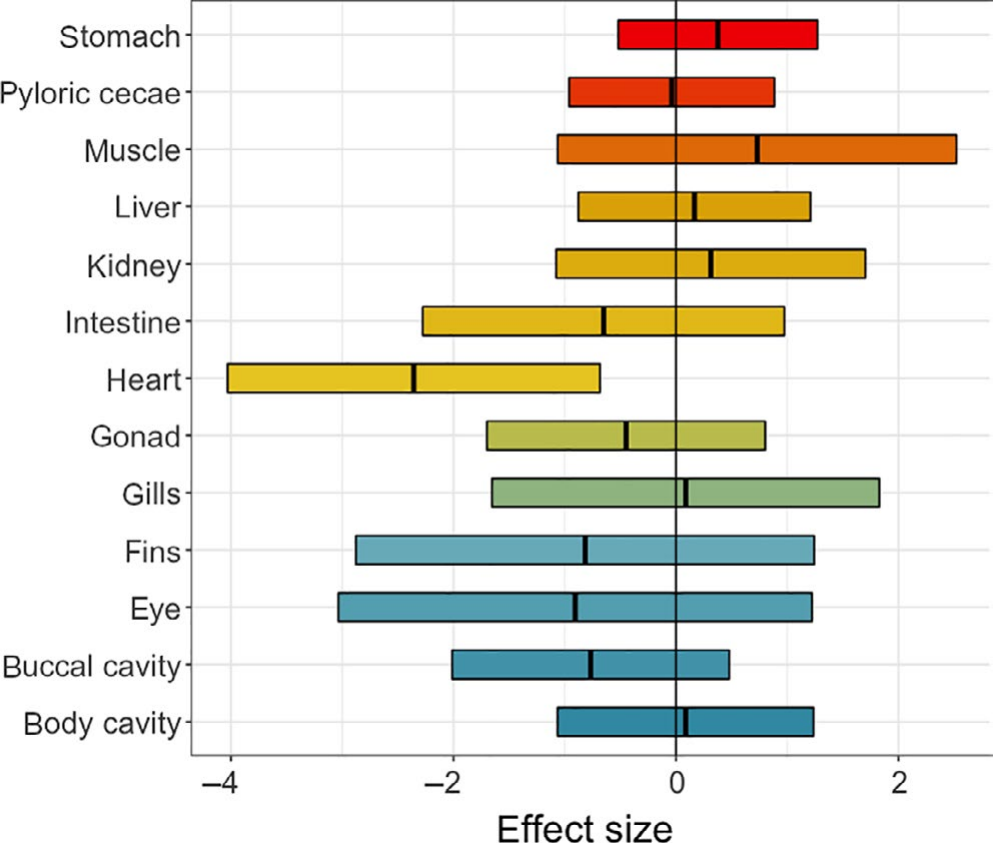
Meta‐regression estimates for the effects of preservation detectability across the various locations in which parasites are found within the host. Positive values indicate higher abundance in the preserved treatment, while negative values indicate higher abundance in the control group. Estimates are shown with 95% confidence intervals

Using the GLMM model estimates (from *Comparing abundance of each parasite taxon between treatments*, above), we ran a meta‐regression model to determine whether there were consistent effects of preservation on detectability among different host tissues. We found no consistent differences in detectability between treatments for parasites found in the body cavity, fins, gills, stomach, intestine, pyloric cecae, muscle, eyes, kidney, liver, gonads, or buccal cavity. However, we did find that parasite detectability was lower in the preservation treatment relative to the control group for parasites found in the heart (Table 3b and Figure 5).

## 4 DISCUSSION

Natural history collections might contain a wealth of information about historical parasite populations, but before we can use these data, they require rigorous validation. We tested whether the fluid‐preservation process alters the detectability of parasites and assessed whether estimates of parasite abundance from natural history collections would be biased by parasite life history stage, taxon, or the host tissue infected. Overall, we found that there were few differences in parasite abundance and distribution between the preservation treatment and control group, with a handful of parasite taxa and host tissues where caution is warranted in interpreting abundance from natural history specimens.

Across the 27 host–parasite pairs, we only found significant differences between the preservation treatment and control group in three pairs. Among these, the direction of the effect was mixed, with some parasites displaying elevated abundances in the preservation treatment relative to the control group and some the reverse. We found that adult hemiuridean trematodes had decreased detectability in the preservation treatment compared to the control group. We noticed that preservation binds host intestinal contents, complicating the detection of adult trematodes in the intestinal lumen. Compared to the intestinal contents of control fish, contents from preserved fish often had to be broken apart manually with forceps instead of by agitation. Larval *Pseudoterranova* sp. nematodes were also less abundant in the preservation treatment than in the control group. Here, the decline in detectability may arise because preservation reduces the color difference between parasite and host tissues, making it more difficult to detect these parasites by transmitted light. For adult *Cucullanus* sp. nematodes, we found that detection was enhanced in the preservation treatment relative to the control group, which might arise because preservation can stiffen nematodes, making them easier to distinguish from intestinal contents. With our sample sizes, we were only able to reliably detect significant differences that had an effect size >0.5. Therefore, our analysis is limited in its ability to detect small differences between treatments.

While there were few significant differences between treatments across parasite taxonomic group and life stage, we were also interested to know whether there were differences in detectability across host organs. We found that, in general, there were few systematic biases in detectability across host tissues, with the only exception being the heart. Parasites from the heart had decreased detectability after preservation. However, we were only able to model a single parasite from the heart, *Pseudoterranova* spp., and thus would caution that further examinations of other fish species are needed to conclusively claim that parasite detectability is decreased in the heart after fixation. For now, a conservative approach would be to consider natural history specimens an unreliable source of information concerning the past abundance of parasites that infect the heart.

Our study is taxonomically limited by the fact that there are certain parasitic taxa that we did not detect or did not detect in sufficient abundance, including ectoparasites such as crustaceans and monogeneans. We also did not screen for the presence of nonmetazoan taxa, such as protozoan, viral, or bacterial parasites. In a study comparing several preservation techniques (fresh, frozen, formaldehyde, and ethanol), it was found that monogenean abundance was reduced by various methods of preservation (Kvach et al., 2018). Preservation‐associated declines in abundance for monogeneans are expected because monogeneans do not have a hard cuticle or outer cyst wall, as nematodes or encysted parasites do, and are ectoparasitic, and this would make them more subject to dehydration during the preservation process. For crustaceans, we would not expect a decline in abundance, since crustaceans have a hard exoskeleton and would be less subject to dehydration and destruction from the preservation process. However, many crustaceans are mobile ectoparasites and could become dislodged during specimen preparation and transfer between preservation fluids. According Kvach et al. (2018), there were no differences in abundance of crustaceans among any of the treatments, thus supporting the hypothesis that crustacean abundance from natural history collections might accurately reflect actual abundance.

We used frozen fish in our experiments because this is the most common state of fish at the time they are accessioned into the UWFC (i.e., it is common for fish to be frozen at sea before thawing, fixation, preservation, and accessioning). The act of freezing a fish is known to reduce the detectability of many parasites, especially protozoa (Kvach et al., 2018). We chose to freeze our fish to accurately reflect the actual process of preservation as it occurs in a major ichthyology collection. A useful extension of this work would be to compare fresh, frozen, and preserved fish to determine how this other component of the natural history collection preservation process could influence the detectability of parasites.

Our experiment addresses the most common method of preservation currently used in fish collections, where the amount of time each specimen spends in fixative is a function of its body size. However, the length of the period of immersion in each fixative or preservative could influence parasite detectability. We sought to understand whether the protocols typically used in natural history collections reduced parasite detectability, and so an investigation of the influence of immersion time in each fixative was beyond the scope of this study. We encourage others to pursue the question of whether the choices made by natural history collection personnel during the fixation process (e.g., how long to leave a specimen in formalin) influence parasite detectability. We also note that formaldehyde was discovered in 1859 (Butlerow, 1859) and may only have been in common use for fixing specimens starting around 1900 (Fox, Johnson, Whiting, & Roller, 1985; Simmons, 1991); specimens collected prior to this date are likely to have been fixed in ethanol, which achieves less complete tissue preservation (Pequignot, Sarot, Duranthon, Pensel, & Carillo‐Baraglioi, 2011). For specimens fixed in anything other than formalin (i.e., formaldehyde diluted with water and buffered), parasite detectability is probably reduced, limiting the temporal scope across which parasitological information can be reliably extracted from natural history collections (Figure 1).

In our experiment, fish were dissected after three to 10 days of ethanol storage (i.e., the minimum sufficient time to ensure that host tissues were saturated with ethanol). In contrast, some specimens in natural history collections have been stored in ethanol for over 100 years (Harmon et al., 2019). Although it is our impression, based on years of experience in fish collections (KM and LT), that the vast majority of physical changes in specimens occur during the fixation and preservation process, long‐term storage in ethanol could further alter the detectability of parasites and result in a loss of information. For example, ethanol might continue to slowly dehydrate the sample over time, warping parasite tissues and making them less recognizable. If fixatives do not fully penetrate the specimen, fixation might be less complete in the interior of fish, leading to decomposition of endoparasites during the course of storage. This could be exacerbated by the action of gut enzymes for intestinal parasites. Our experiment does not address the changes in parasite detectability that might occur during long‐term storage; the way to assess this would be to repeat the procedure used herein, but with multiple time points of dissection over a long time span. Our group has initiated this experiment by holding experimentally preserved fish that we will dissect at multiple time points over the next decade to determine the effects of long‐term storage in ethanol on parasite detectability.

Although our study suggests that a fixed fish specimen accurately reflects the parasite burden of the fish at the time of its fixation, it does not address the potential for systematic bias in the choice of which fish to preserve, which could have a substantial influence on estimates of parasite abundance. Some collections may receive exhaustive fish samples from a specific location year after year; for example, the Eulachon and Walleye Pollock we dissected came from research trawls in which the entire catch was retained. In this case, the collections represent an unbiased sample of natural populations and should accurately reflect natural populations. On the other hand, many collections are the result of more haphazard collections, or collections motivated by past research projects or curator interests. This can introduce artifacts as collectors and collections staff select which fish to be cataloged, which might either favor parasitism or disfavor parasitism (Harmon et al., 2019). For example, curators might choose not to catalog a fish that is visibly parasitized for aesthetic reasons (i.e., selecting ideal specimens for taxonomic research) or might intentionally choose to catalog it as a curiosity. These biases can also change through time as personnel and research practices change. Addressing this potential source of bias requires discussions with collections staff to understand the history of policies and practices in the natural history collection of interest.

## 5 CONCLUSIONS

Natural history collections contain tens of millions of preserved fishes, representing a treasure trove of information on the health of populations and communities (Harmon et al., 2019; Singer, Ellis, & Page, 2020). Our results suggest that the fluid‐preservation approach used by ichthyological collections has little effect on the detectability of parasites, but whether subsequent long‐term storage affects parasite detectability remains an open question. If future studies find that long‐term storage does not alter estimates of parasite abundance, this should allow ecologists to confidently use parasitological dissection of natural history specimens for reconstructing long‐term parasite change over the past 100 years or more. “Long‐term” empirical datasets on fish parasites have an average length of 12.4 years, so use of natural history specimens could significantly broaden the range of dates for which parasitological information is available, and many previously intractable questions in disease ecology will be within our reach using this new approach. Most importantly, the long‐term perspective provided by this approach will allow us to evaluate contemporary parasite burdens in their historical context.

## CONFLICT OF INTEREST

The authors report no potential conflicts of interest.

## AUTHOR CONTRIBUTION

**Evan A. Fiorenza:** Conceptualization (supporting); Data curation (lead); Formal analysis (lead); Investigation (lead); Methodology (supporting); Project administration (supporting); Software (lead); Visualization (equal); Writing‐original draft (equal); Writing‐review & editing (supporting). **Katie L. Leslie:** Conceptualization (supporting); Data curation (supporting); Investigation (supporting); Methodology (supporting); Writing‐original draft (supporting); Writing‐review & editing (supporting). **Mark E. Torchin:** Conceptualization (supporting); Methodology (supporting); Writing‐original draft (supporting); Writing‐review & editing (supporting). **Katherine P. Maslenikov:** Conceptualization (supporting); Methodology (supporting); Project administration (supporting); Resources (equal); Supervision (supporting); Writing‐original draft (supporting); Writing‐review & editing (supporting). **Luke Tornabene:** Conceptualization (supporting); Methodology (supporting); Project administration (supporting); Resources (equal); Supervision (supporting); Writing‐original draft (supporting); Writing‐review & editing (supporting). **Chelsea L. Wood:** Conceptualization (lead); Data curation (supporting); Formal analysis (supporting); Funding acquisition (lead); Investigation (supporting); Methodology (lead); Project administration (supporting); Resources (equal); Software (supporting); Supervision (lead); Visualization (equal); Writing‐original draft (equal); Writing‐review & editing (equal).

### Open Research Badges

This article has been awarded Open Materials and Open Data Badges. All materials and data are publicly accessible via the Open Science Framework at: https://doi.org/10.5061/dryad.v9s4mw6s6; https://github.com/wood-lab/Fiorenza_et_al_2020_Ecol_Evol.

## Supporting information

Table S1

## Data Availability

The data that support the findings of this study are openly available in the Dryad Digital Repository at https://doi.org/10.5061/dryad.v9s4mw6s6. The code used to produce the statistical results reported herein is openly available via GitHub at https://github.com/wood-lab/Fiorenza_et_al_2020_Ecol_Evol.
