# Supplementary material for: Fluid preservation causes minimal reduction of parasite detectability in fish specimens: A new approach for reconstructing parasite communities of the past?
Source: Ecol Evol. 2020 Jun 15;10(13):6449–60. doi: 10.1002/ece3.6379 (PMC7381554; doi:10.1002/ece3.6379)
Supplement: Supplementary file 1 — Table S1 [file ECE3-10-6449-s001.docx]

**Supplementary Table S1.** Studies included in a meta-analysis aimed at estimating the temporal length of fish parasite datasets described as “long-term” in the literature. These studies were extracted from ISI Web of Science with the search string TS = ((long-term OR long term) AND parasite AND fish) on 30 March 2020, which returned 285 potential articles. We then screened the titles and abstracts of these articles for relevance to our question. To be included, each study had to meet the following criteria: (1) contain annual or nearly annual (i.e., >50% of temporal period had observations) observations of parasite abundance or prevalence in fishes (i.e., a study comparing two observations of parasite abundance 40 years apart is not eligible), (2) describe observations, not the results of an experimental manipulation, (3) contain data on parasitic infections of marine or freshwater fishes, not invertebrates, (4) contain data that do not arise from parasitological dissection of natural history collections, (5) include the phrase “long term” or “long-term” in the title or abstract as a descriptor of the study (e.g., “long-term speciation” would not meet the criteria), and (6) contain data from wild fish, not aquaculture-reared fish. This winnowing yielded the 25 articles shown below. From each of these articles, we extracted the length of the dataset in years.

| **Authors** | **Year** | **Title** | **Journal** | **Volume** | **Page range** | **Length of dataset in years** |
| --- | --- | --- | --- | --- | --- | --- |
| Negreiros LP; Florentino AC; Pereira, FB; Tavares-Dias M | 2019 | Long-term temporal variation in the parasite community structure of metazoans of *Pimelodus blochii* (Pimelodidae), a catfish from the Brazilian Amazon | *Parasitology Research* | 118 | 3337-47 | 6 |
| Hoshino EM; Tavares-Dias M | 2019 | Temporal and seasonal variations in parasites of *Metynnis lippincottianus* (Characiformes: Characidae), a host from the eastern Amazon (Brazil) | *Journal of Natural History* | 53 | 2723-36 | 6 |
| Sikkel PC; Richardson MA; Sun D; Narvaez P; Feeney WE; Grutter AS | 2019 | Changes in abundance of fish-parasitic gnathiid isopods associated with warm-water bleaching events on the northern Great Barrier Reef | *Coral Reefs* | 38 | 721-30 | 19 |
| Reyda FB; Pommelle CP; Doolin ML | 2019 | Asian fish tapeworm (*Schyzocotyle acheilognathi*) found in New York State for the first time after a long-term fish-parasite survey | *Comparative Parasitology* | 86 | 108-  13 | 11 |
| Heins DC; Moody KN; Miller S | 2019 | Are solo infections of the diphyllobothriidean cestode *Schistocephalus solidus* more virulent than multiple infections? | *Parasitology* | 146 | 97-  104 | 11 |
| Gavrilov AL; Gos'kova OA | 2018 | The long-term dynamics of parasite infection in coregonids with different food specializations | *Russian Journal of Ecology* | 49 | 548-  53 | 25 |
| Shukhgalter OA; Lidvanov VV | 2018 | Long-term dynamics of mesozooplankton communities and parasite fauna of the European pilchard (*Sardina pilchardus* Walbaum, 1792) from the coastal zone of Morocco in 1994-2011 | *Zhurnal Obshchei Biologii* | 79 | 108-  23 | 17 |
| **Authors** | **Year** | **Title** | **Journal** | **Volume** | **Page range** | **Length of dataset in years** |
| Klapper R; Bernreuther M; Wischnewski J; Klimpel S | 2017 | Long-term stability of *Sphyrion lumpi* abundance in beaked redfish *Sebastes mentella* of the Irminger Sea and its use as biological marker | *Parasitology Research* | 116 | 1561-  72 | 4 |
| Young RE; Maccoll ADC | 2017 | Spatial and temporal variation in macroparasite communities of three-spined stickleback | *Parasitology* | 144 | 436-  49 | 8 |
| Kuhn JA; Knudsen R; Kristoffersen R; Primicerio R; Amundsen P-A | 2016 | Temporal changes and between-host variation in the intestinal parasite community of Arctic charr in a subarctic lake | *Hydrobiologia* | 783 | 79-  91 | 20 |
| May-Tec AL; Pech D; Aguirre-Macedo ML; Lewis JW; Vidal-Martinez VM | 2013 | Temporal variation of *Mexiconema cichlasomae* (Nematoda: Daniconematidae) in the Mayan cichlid fish *Cichlasoma urophthalmus* and its intermediate host *Argulus yucatanus* from a tropical coastal lagoon | *Parasitology* | 140 | 385-  95 | 8 |
| Bentley KT; Burgner RL | 2011 | An assessment of parasite infestation rates of juvenile sockeye salmon after 50 years of climate warming in southwest Alaska | *Environmental Biology of Fishes* | 92 | 267-  73 | 12 |
| Shadrin AM; Pavlov DS; Kholodova MV | 2010 | Long-term dynamics of infection of fish eggs and larvae with the endoparasite *Ichthyodinium* sp. (Dinoflagellata) in Nha Trang Bay, Vietnam | *Fish Pathology* | 45 | 103-  8 | 17 |
| Vethaak AD; Jol JG; Pieters JPF | 2009 | Long-term trends in the prevalence of cancer and other major diseases among flatfish in the southeastern North Sea as indicators of changing ecosystem health | *Environmental Science and Technology* | 43 | 2151-58 | 15 |
| Ozer, Ahmet | 2007 | Metazoan parasite fauna of the round goby *Neogobius melanostomus* Pallas, 1811 (Perciformes: Gobiidae) collected from the Black Sea coast at Sinop, Turkey | *Journal of Natural History* | 41 | 483-  92 | 1 |
| Schabuss M; Kennedy CR; Konecny R; Grillitsch B; Schiemer F; Herzig A | 2005 | Long-term investigation of the composition and richness of intestinal helminth communities in the stocked population of eel, *Anguilla anguilla*, in Neusiedler See, Austria | *Parasitology* | 130 | 185-  94 | 8 |
| Zander, CD | 2005 | Four-year monitoring of parasite communities in gobiid fishes of the southwest Baltic - III. Parasite species diversity and applicability of monitoring | *Parasitology Research* | 95 | 136-  44 | 4 |
| **Authors** | **Year** | **Title** | **Journal** | **Volume** | **Page range** | **Length of dataset in years** |
| Schmidt V; Zander S; Korting W; Steinhagen D | 2003 | Parasites of the flounder *Platichthys flesus* (L.) from the German Bight, North Sea, and their potential use in ecosystem monitoring - A. Infection characteristics of potential indicators species | *Helgoland Marine Research* | 57 | 236-51 | 6 |
| Knudsen R; Amundsen PA; Klemetsen A | 2002 | Parasite-induced host mortality: Indirect evidence from a long-term study | *Environmental Biology of Fishes* | 64 | 257-  65 | 12 |
| Kennedy CR; Shears PC; Shears JA | 2001 | Long-term dynamics of *Ligula intestinalis* and roach *Rutilus rutilus*: A study of three epizootic cycles over thirty-one years | *Parasitology* | 123 | 257-  69 | 31 |
| Anshary H; Ogawa K; Higuchi M; Fujii T | 2001 | A study of long-term changes in summer infection levels of Japanese flounder *Paralichthys olivaceus* with the monogenean *Neoheterobothrium hirame* in the central Sea of Japan, with an application of a new technique for collecting small parasites from the gill filaments | *Fish Pathology* | 36 | 27-  32 | 5 |
| Bennett SN; Adamson ML; Margolis L | 1998 | Long-term changes in parasites of sockeye salmon (*Oncorhynchus nerka*) smolts | *Canadian Journal of Fisheries and Aquatic Sciences* | 55 | 977-  86 | 41 |
| Amundsen PA; Kristoffersen R; Knudsen R; Klemetsen A | 1997 | Infection of *Salmincola edwardsii* (Copepoda: Lernaeopodidae) in an age-structured population of Arctic charr - A long-term study | *Journal of Fish Biology* | 51 | 1033-  46 | 8 |
| McKeown CA; Irwin SWB | 1997 | Accumulation of *Diplostomum* spp. (Digenea: Diplostomatidae) metacercariae in the eyes of 0+ and 1+ roach (*Rutilus rutilus*) | *International Journal for Parasitology* | 27 | 377-  80 | 3 |
| MacKenzie K | 1987 | Long-term changes in the prevalence of two helminth-parasites (Cestoda, Trypanorhyncha) infecting marine fish | *Journal of Fish Biology* | 31 | 83-  87 | 11 |
